# Photoacoustic Characterization of TiO_2_ Thin-Films Deposited on Silicon Substrate Using Neural Networks

**DOI:** 10.3390/ma16072865

**Published:** 2023-04-04

**Authors:** Katarina Lj Djordjević, Dragana K. Markushev, Marica N. Popović, Mioljub V. Nesić, Slobodanka P. Galović, Dragan V. Lukić, Dragan D. Markushev

**Affiliations:** 1 “Vinča” Institute of Nuclear Sciences, National Institute of the Republic of Serbia, University of Belgrade, P.O. Box 522, 11000 Belgrade, Serbia; 2 Institute of Physics Belgrade, National Institute of the Republic of Serbia, Pregrevica 118, University of Belgrade, Zemun, 11080 Belgrade, Serbia

**Keywords:** thin-film, TiO_2_, photoacoustic, artificial neural networks, thermal diffusion, thermal expansion, inverse problem

## Abstract

In this paper, the possibility of determining the thermal, elastic and geometric characteristics of a thin TiO_2_ film deposited on a silicon substrate, with a thickness of 30 μm, in the frequency range of 20 to 20 kHz with neural networks were analysed. For this purpose, the geometric (thickness), thermal (thermal diffusivity, coefficient of linear expansion) and electronic parameters of substrates were known and constant in the two-layer model, while the following nano-layer thin-film parameters were changed: thickness, expansion and thermal diffusivity. Predictions of these three parameters of the thin-film were analysed separately with three neural networks. All of them together were joined by a fourth neural network. It was shown that the neural network, which analysed all three parameters at the same time, achieved the highest accuracy, so the use of networks that provide predictions for only one parameter is less reliable. The obtained results showed that the application of neural networks in determining the thermoelastic properties of a thin film on a supporting substrate enables the estimation of its characteristics with great accuracy.

## 1. Introduction

The photoacoustic effect is the effect of the appearance of sound in the gaseous environment of a sample that is illuminated. This effect was discovered by A. G. Bell in 1880 [1], and explained by A. Rosencwaig almost 100 years later, in 1975 [2,3,4]. If the sample is exposed to the effect of electromagnetic radiation, part of the excitation energy is absorbed and part of the absorbed energy is transformed into heat through a non-radiative de-excitation relaxation process. This process is also called the photothermal effect. The heated sample generates a disturbance of the thermodynamic equilibrium with the environment and, as a result, there is a fluctuation of pressure, density and temperature in both the sample itself and in its gaseous surrounding. These fluctuations affect the appearance of several phenomena that can be detected in different ways [4]. Numerous non-destructive methods, known as photothermal methods, based on the recording of these phenomena, have been developed in the last half-century and are increasingly used for the characterization of various materials, electronic devices, sensors, biological tissues, etc. Pressure fluctuations are, in fact, a sound signal, the so-called photoacoustic effect, which can be detected using piezoelectric or ultrasonic sensors as well as a microphone [5,6,7,8,9,10,11]. The gas microphone photoacoustic was the first developed and today is one of the most widespread experimental techniques. The implementation of this measuring technique with a cell of minimal volume, proposed in the early 1980s, ensures that acoustic losses are attenuated as much as possible in detection.

In the last decade, TiO_2_ has had a wide range of applications in coatings, medicines, plastics, food, inks, cosmetics, and textiles. In the form of thin-film, TiO_2_ has been used for a great variety of applications, including photocatalytic degradation of organic pollutants in water as well as in air, dye-sensitized solar cells, anti-fogging, super hydrophilic, micro- and nano-mechanical sensors, etc. [12,13,14,15]. To be able to measure the physical properties of such thin films, it is usually necessary to deposit such a film on a thicker wafer.

The analysis of thin-films on substrates has always been a challenge for photoacoustic because film thicknesses ranges from a few tens to several hundred nanometres. Depending on the thickness of the substrate (usually more than tens of microns), such film thicknesses are usually at the limit of experimental detection [16,17,18,19]. This means, for example, that the differences in the amplitude of the photoacoustic signals (PAS), generated by a two-layer sample (substrate + thin-film) in the case where only the thickness of the film is changed, are extremely small [20,21,22,23,24,25,26,27,28]. The analysis of such two-layer samples is also theoretically demanding.

For photoacoustic measurements to be used in the characterization of materials, it is necessary to develop a theoretical model that well describes all the processes involved in the formation of the measured signal: the process of absorption and its conversion into heat, which depends on the optical properties of the sample, the processes of heat conduction and sound propagation, which depend on the thermal and elastic properties of the sample and the thermodynamic pressure change in the gaseous environment of the sample, that is, the sound signal formed by the heated sample and recorded by a microphone. The inverse solution of the photoacoustic problem is essentially a multi-parameter fitting of the sample properties based on the developed model, which should lead to the best matching of the theoretical model with the experimentally measured signal. Since it is a multi-parameter problem, which is also a non-linear and ill-posed problem of mathematical physics due to the limited measurement range, the inverse photoacoustic problem is still the subject of intensive research, especially in the case of multi-layered structures or semiconductors where an increased number of parameters influence the recorded increase in signals (in semiconductors, photogenerated carriers affect the recorded signal. In multi-layered structures, the same processes occur in all layers, but they are controlled by properties of each layer). This makes solving the inverse photoacoustic problem extremely difficult.

Recently, machine learning has been introduced to solve the inverse photoacoustic problem. The achieved results are encouraging because they show that the application of neural networks allows a very high accuracy of the multi-parameter fitting.

The earlier developed procedure based on neural networks [10,11,29,30,31] for processing of experimentally recorded photoacoustic signals of silicon samples by the open photoacoustic cell [32,33,34,35] shows effective recognition and removal of instrumental influence [33,34,35,36,37,38,39,40], and, consequently, provides a detailed and precise characterization of the sample [41,42,43,44,45,46]. On the other hand, a very thin TiO_2_ layer (nano-layer) is easily deposited in a silicon substrate. Therefore, we selected a well-photoacoustically characterized silicon sample as the substrate, open photoacoustic cell photoacoustic set-up for measurement, and neural networks for solving the inverse PA photoacoustic problem and determining the thin-film’s properties.

In order to avoid additional normalizations and the calculation of effective values, we resorted to the use of the two-layer model for determining thin-film parameters where the properties of the silicon substrate are known [21,47,48,49,50,51,52,53,54,55,56,57,58,59,60,61,62]. Neural networks were formed for the analysis of photoacoustic signals generated from the Si substrate and the TiO_2_ thin-film system.

Based on previous experiences in PAS processing, we expected that they would recognize differences in signals caused by only changing film parameters (thickness, thermal diffusivity, coefficient of thermal expansion). We also expected that neural networks can determine the specified parameters of TiO_2_ thin-film with satisfactory accuracy and reliability. To do this, we created a relatively small database of photoacoustic signals for training and four types of networks; three of them serve as the individual predictions of only one parameter of the film, and the fourth, which serves as the prediction of all three parameters simultaneously.

In Section 2, a brief description of the theoretical model for the PAS measured on a two-layer structure is given. In Section 3, the network architecture used in the work is explained. Section 4 explains in detail how the base upon which the networks were trained and tested was formed. In Section 5, the results are given and discussed. In the end, the most important conclusions were drawn. The obtained results show that the application of neural networks in determining the thermoelastic properties of a thin-film on a supporting substrate enables the estimation of thin-film characteristics with great accuracy.

## 2. Experimental Procedure

**Figure 1:**
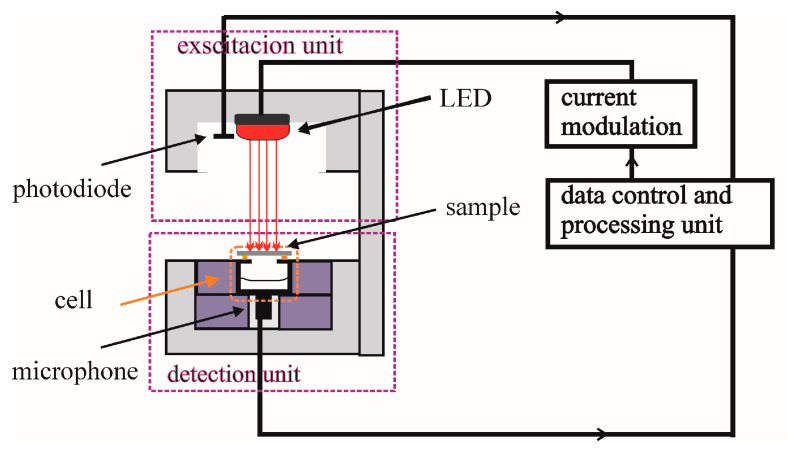
Open-cell experimental set-up in transmission configuration.

The open-cell experimental photoacoustic set-up in a transmission configuration is illustrated in Figure 1. Excitation is performed by a low-power 10 mW laser/LED (XL7090-RED, RF Communication Electronic Technology Co., Ltd., Xiamen, China) diode regulated by a frequency generator in the range of 20 Hz to 20 kHz and which illuminates the sample with a red light of a wavelength of 660 nm with a distance that ensures homogeneous (uniform) surface illumination. Illumination control is performed by a sensitive photodiode (BPW34 Vishay Telefunken).

After absorption and excitation of the sample structural units, thermal energy is released through a non-radiative relaxation process, causing changes in the temperature profile of the sample. Periodic excitation generates a periodic change in the temperature distribution of the sample, which leads to periodic change in the pressure in the microphone hole that serves as a photoacoustic cell [32]. The sample is placed directly on the photoacoustic cell. The pressure changes are very small, ~10^−6^ bar, but the MC60 microphone, due to its sensitivity, detects their amplitudes and phase deviations from excitation optical signals recorded by the photodiode at each modulation frequency. The photoacoustic response is finally given in an amplitude-phase characteristic in a wide range of frequencies, from 10 Hz to 20 kHz.

The open photoacoustic cell [32], is formed so that the inside of the microphone represents a cell. Thus, the measurement takes place with a minimum volume, which enables the recording of weak sound signals. In the measuring set-up from Figure 1, the computer sound card (Intel 82,801 Ib/ir/ihhd) is used for making the lock-in amplifier. The sampling of the modulation frequencies is programmed in a regular logarithmic equidistant step. The photoacoustic response recorded in this way is suitable for the analysis of silicon samples up to 1 mm thick, with layers of thin-films with a thickness of up to several 100 nm, or the analysis of thin layers of multilayer structures.

**Figure 2:**
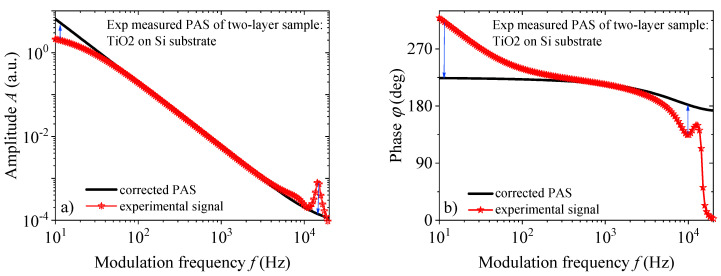
Frequency dependence of (**a**) amplitude and (**b**) phase of experimentally measured photoacoustic signal TiO_2_ placed on Si substrate (red asterisk) and the corresponding amplitude and phase of the photoacoustic signal *δp*_total_(*f*) (black line), correction on the instrument input (blue arrows).

One of the problems of photoacoustics is that the entire measurement frequency range is most often not used due to the influence of the accompanying measurement instrumentation in the low and high-frequency ranges. The influence of the used instruments is reflected in the fact that the amplitude of the photoacoustic signal of the sample is distorted in the low and high frequency parts, and the phase shifts its position, as is shown in Figure 2. With the developed methodology of removing the instrumental influence [35,36,37,38,39,40], from the microphone to the accompanying electronics, it was shown that it is possible from the recorded photoacoustic response *S*(*f*) to obtain the photoacoustic signal *δp*_total_(*f*), with a wide frequency range of 20 to 20 kHz, which can be used for further precise characterization [36,37,38,39,40]. The instrumental influence in the photoacoustic experiment can be described by the transfer function *H*(*f*), which distorts the photoacoustic signal of the sample *δp*_total_(*f*), in the following way:

(1) $$S\left(f\right)=\delta p_{\text{total}}\left(f\right)H\left(f\right)$$

(2) $$H\left(f\right)=H_{\text{total}}^{\text{e}}\left(f\right)H_{\text{total}}^{\text{a}}\left(f\right)$$

The form of the function $H\left(f\right)=H_{\text{total}}^{\text{e}}\left(f\right)H_{\text{total}}^{\text{a}}\left(f\right)$ used for filtering in the low-frequency part represents the transfer functions, which characterize the influences of the microphone and accompanying electronics:

(3) $$H_{\text{total}}^{\text{e}}\left(f\right)=-\frac{\omega \tau_{c1}}{\left(1+i\omega \cdot \tau_{c1}\right)}\cdot \frac{\omega \;\tau_{c2}}{\left(1+i\omega \cdot \tau_{c2}\right)}\;,$$

where time constants are *τ_c_*_1_ = (2π*f_c_*_1_)^−1^ and *τ_c_*_2_ = (2π*f_c_*_2_)^−1^, the attenuation factor is *δj* (*j* = *c*3,*c*4), the peak frequency is denoted by ω*_c_*_3_ and cut- by ω*_c_*_4_ (ω = 2π*f*) (blue arrows, Figure 2). The function of form $H_{\text{total}}^{\text{a}}\left(f\right)$ is used for filtering in the high-frequency part. It is a combination of second-order transfer functions:

(4) $$H_{\text{total}}^{\text{a}}\left(f\right)=\frac{\omega_{c3}^{2}}{\omega_{c3}^{2}+\delta_{c3}i\omega -\omega^{2}}+\frac{\omega_{c4}^{2}}{\omega_{c4}^{2}+\delta_{c4}i\omega -\omega^{2}}\;,$$

The correction procedure of the experimentally recorded photoacoustic response of multilayer samples produces a signal that can be further analyzed using a theoretical model and all frequency ranges of the measurement.

## 3. Theoretical Background

**Figure 3:**
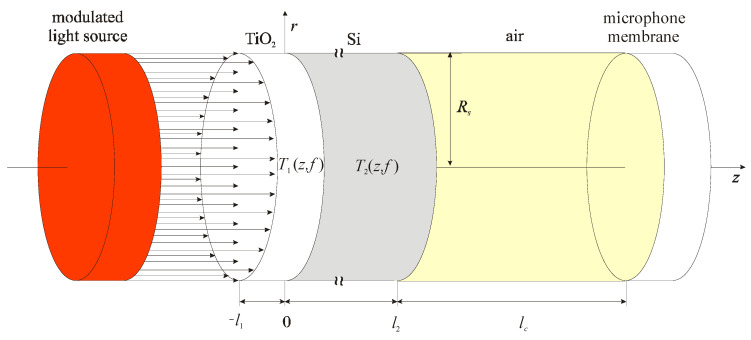
The simplest scheme of the two−layer sample irradiated by modulated light source. *l*_1_ and *l*_2_ (*l*_1_ << *l*_2_) are the thicknesses of the thin−film (TiO_2_) and substrate (Si), respectively. *R*_s_ is the sample radius, *T*_1_(*z,f*) and *T*_2_(*z,f*) is the temperature distribution in the thin-film and substrate.

Using uniform illumination of the two-layer sample (Figure 3) with a modulated light source, the electromagnetic radiation is absorbed and produces a periodic change in the thermal state of both the thin-film and the substrate. The layer of TiO_2_ is considered dielectric because there is no effect of photogenerated charge carriers due to the larger energy gap of TiO_2_ in comparison to the photon energy of the exciting beam, while the photogenerated charge carriers affect the temperature profile of the silicon substrate *T*_2_(*z,f*). Temperature changes of the non-illuminated side of the sample *T*_2_(*l,f*) and the temperature gradient between the illuminated and non-illuminated sides of the sample causes the change in the thermodynamic state in the air behind the sample. Such fluctuations create three different components of sound that result from thermal transfer from the elastic bending of the sample (composite piston theory) that the microphone detects as a total photoacoustic signal *δp*_total_(*f*), defined as [10,11,21,30,63,64,65,66]:

(5) $$\delta p_{\mathrm{total}}\left(f\right)=\delta p_{\mathrm{TD}}\left(f\right)+\delta p_{\mathrm{TE}}\left(f\right)+\delta p_{\mathrm{PE}}\left(f\right),$$

where *f* is the modulation frequency, and *δp*_TD_(*f*), *δp*_TE_(*f*) and *δp*_PE_(*f*) are the thermodiffusion (TD), thermoelastic (TE) and plasmaelastic (PE) photoacoustic signal components, respectively. The thermodiffusion component arises as a result of periodic heating of the non-illuminated surface of the sample, which periodically heats the air layer, causing it to periodically expand and contract. The periodic expansion and contraction of the air layer create a disturbance that is detected by the microphone. The thermoelastic component arises due to the temperature gradient between the illuminated and non-illuminated sides of the sample, which leads to the bending of the sample. Due to the modulation of the illumination, the bending is periodic, which pushes the pressure in the air that is detected by the microphone. The plasmaelastic component is caused by the photogeneration of carriers due to illumination, which leads to the additional bending of the sample, caused by a concentration gradient of charge carrier that pushes the pressure in the air which is then detected by the microphone. These components can be written as [10,11,21,30,63,64,65,66]:

(6) $$\delta p_{\mathrm{TD}}(f)=\frac{p_{0}\gamma_{g}}{\sigma_{g}l_{c}}\frac{T_{2}(l_{2},f)}{T_{0}},$$

(7) $$\delta p_{c}\left(f\right)=\frac{\gamma_{g}p_{0}}{V_{0}}\int_{0}^{R_{s}}2\pi rU_{z,c}\left(r,z\right)dr\;c=\mathrm{TE},\;\mathrm{PE}$$

where *γ_g_* is the adiabatic constant, *p*_0_ and *T*_0_ represent the standard pressure and temperature of the air in the microphone, $\sigma_{g}=\left(1+i\right)/\mu_{g}$, $\mu_{g}$ is the thermal diffusion length of the air, *l_c_* is the photoacoustic cell length, *T*_2_(*l*_2_,*f*) is the dynamic temperature variation at the substrate rear (non-illuminated) surface [10,11,21,30,63,64,65,66] (see Appendix A), *V*_0_ is the open photoacoustic cell volume and *U_z,c_*(*r*,*z*) is the sample displacement along the *z*-axes (see Appendix B).

The total photoacoustic sound signals *δp*_total_(*f*), (Equation (5)) are usually represented using its amplitudes *A*(*f*) and phases *φ*(*f*). Therefore, *δp*_total_(*f*), can be written as a complex number in the form:

(8) $$\delta p_{\mathrm{total}}\left(f\right)=A\left(f\right)e^{i\varphi \left(f\right)},$$

where *i* is the imaginary unit. The theoretically calculated photoacoustic signal *δp*_total_(*f*) is comparable to the experimentally recorded amplitude and phase from which the instrumental influence has been removed (Equations (1)–(4)). Thus, by analytically developing the model and numerical simulations, a standard method can be used for making the base of signals required for neural networks. The application of neural networks in photoacoustics for characterization requires an adjusted value of amplitude in order to be comparable with the values of phase. A formula used for this purpose has a form:

(9) $$A_{scale}\left(f\right)=20\log_{10}A\left(f\right).$$

The theoretically determined photoacoustic signal *δp*_total_(*f*), is compared with the experimentally recorded amplitude and phase, and is used for material characterization.

## 4. Networks Structure

**Figure 4:**
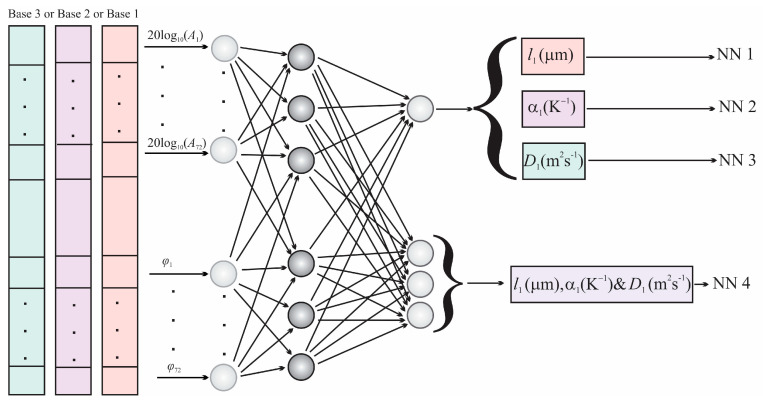
A representation of the structure of a single−layer neural network used for the training and prediction of TiO_2_ thin−film parameters.

The structure of the networks used to characterize the thin-films on the substrate is shown in Figure 4. All networks used in this paper have the same structure: 2 × 72 input neurons (72 amplitudes and 72 phases) and 15 neurons in the hidden layer. The three networks, labeled NN1, NN2 and NN3, have one neuron each in the output layer that serves to predict the *l*_1_, *α_Τ_1__* and *D_T_1__* thin-film parameters, respectively. The network designated as NN4 has three neurons in the output layer that simultaneously predict all three mentioned parameters. The bases formed for the training of the first three networks were made individually (Base 1, Base 2 and Base 3), while the training base NN4 (Base 4) was made by merging all three individual bases [67,68,69,70].

The training process involved neural network training on theoretical signal Bases 1–4, amplitude-phase characteristics and the connection with the parameters of the thin-film, performed by an algorithm that uses statistical models of machine learning that enable prediction, as shown in Figure 4. In the prediction process, thin-film parameters are determined from the test signal or the experimentally recorded photoacoustic signal.

## 5. Formation of the Networks Training Bases

**Figure 5:**
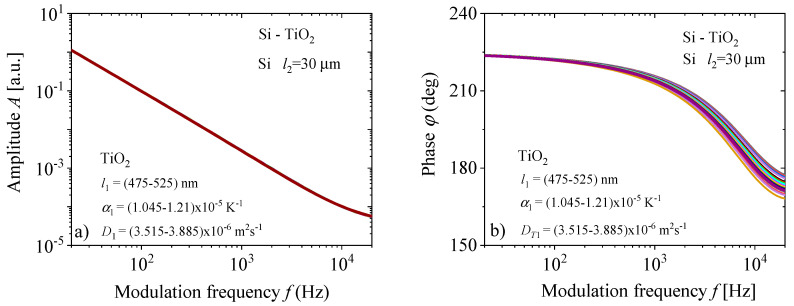
(**a**) Amplitudes, *A*, and (**b**) phases, *φ*, of the two−layer model: TiO_2_ thin−films deposited on the Silicon substrate, obtained by changing parameters of the thin−film, diffusivity *D_T_*_1_, expansion *α_T_*_1_ and thickness *l*_1_.

The accuracy of the neural network largely depends on the selection of the basis for training, testing and validation. The bases have been obtained numerically using Equations (5)–(9). It is assumed that all these signals are generated by the Si substrate and TiO_2_ thin-film two-layer system presented in Figure 3. All bases consist of 41 photoacoustics and one basic. The rest of them were obtained by changing 10% of the TiO_2_ thin-film parameters. The basic parameters as a system property that affects the photoacoustic signal include: geometric (thickness), thermal (thermal diffusivity, coefficient of linear expansion) and electronic, which depend on the level of doping and the purity of Si and the properties of the TiO_2_ thin-film, which are shown in Table 1, with standard temperature and pressure. Base 1 was formed for NN1 training, changing the thickness of TiO_2_ film in the range of *l*_1_ = (475–525) nm with a step of 5 nm. Base 2 was formed for NN2 training, obtained by changing the coefficient of thermal expansion of TiO_2_ film in the range of *α*_1_ = (1.045–1.55) × 10^−5^ K^−1^ with a step of 5 × 10^−8^ K^−1^. Base 3 was formed for NN3 training, changing the thermal diffusivity of TiO_2_ film in the range of *D*_1_ = (3.515–3.885) × 10^−6^ m^2^s^−1^ with a step of 18.5 × 10^−8^ m^2^s^−1^. Base 4 was formed for NN4 training, obtained by collecting 3 × 41 signals from all three previously mentioned bases. Since all bases are very similar, we will show only one of them, Base 4, bearing in mind that, by one photoacoustic signal, we mean two curves presented in the networks: one for amplitude and another for phase (Equation (9) and Figure 5).

By displaying the photoacoustics of a silicon substrate thickness of *l*_2_ = 30 μm, with different applied layers *l*_1_ of TiO_2_ thin-film, it is observed that there is no clear visual difference in the frequency dependence of the amplitudes, *A*, and that the factor of precise characterization by neural networks can be a visible difference in signal phases, *φ*, especially in the range from 10^3^ Hz to 20 kHz, shown in Figure 5. The difference that exists in the phases is sufficient to train neural networks NN1-4 on the amplitude-phase characteristics and to correctly determine the parameters of a thin layer that is two orders of magnitude thinner than the substrate.

## 6. Results and Discussion

**Figure 6:**
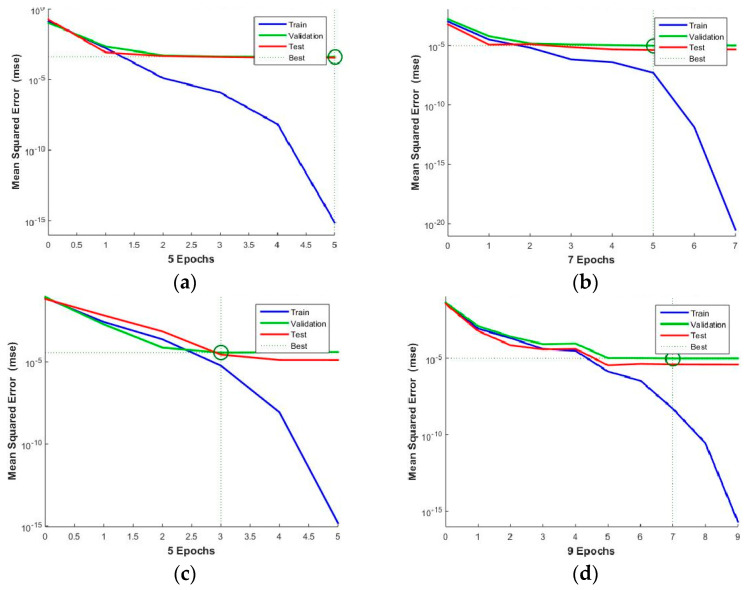
Network training: (**a**) NN1, (**b**) NN2, and (**c**) NN3 for determining the parameters of thickness, expansion, and diffusivity of the TiO_2_ thin−film, respectively, and (**d**) NN4 for determining all three data, simultaneously.

The training results of the NN1-4 neural networks are given in Figure 6a–d, showing the Mean Square Error (MSE) of training, test and validation, depending on the number of epochs, and obtaining the best training performance. From each base for NN1-3 training, four signals were extracted for later testing. In the case of NN4 training, 3 × 4 = 12 signals were also extracted from Base 4 for later testing. Network training interruption is activated by the deviation criterion of Mean Square Error training in relation to validation and testing. The performance achieved by network NN1 is 4.1292 × 10^−4^ in 5 epochs, network NN2 achieved 9.5639 × 10^−6^ in 5 epochs, network NN3 achieved 3.6325 × 10^−5^ in 3 epochs and network NN4 achieved 9.8558 × 10^−6^ in 7 epochs. It can be seen by comparing these values that the best performance was obtained by the NN4 and NN2 networks for determining all three parameters and expansion, respectively. The NN1 network obtained the weakest performance for determining the thin-film thickness parameter.

### 6.1. Networks Testing with In-Step, Out-Step of Photoacoustic Signal

As we said in the previous paragraph, four signals that did not participate in the training were separated from each training base of the NN1-3 networks. A similar thing was carried out with the training base for the NN4 network, from which 12 signals were separated and did not participate in the training. All four networks were tested with these “in-step” signals and the results of such tests are shown in Table 2 and Table 3. Relative error predictions (%) presented in these tables show that the most accurate networks are NN2 for the prediction of *α_T_*_1_ and NN4 for the prediction of *D_T_*_1_.

Our next step is to check the quality of the prediction of neural networks with “out-step” signals—signals outside the training step but within the framework of parameter changes. For this purpose, 12 signals were randomly generated. Four for each changed parameter *l*, *α_T_* and *D_T_* individually. The prediction results for all four networks are given in Table 4 (NN1-3) and Table 5 (NN4). It is interesting to note that the NN1 network gives the worst prediction of sample thickness, while the NN4 network gives relatively satisfactory predictions for all three parameters.

### 6.2. Networks Testing with Experimental Signals

The final part of our analysis is to test the ability to predict our networks on experimental signals. For this purpose, we measured, by the standard method of an open photoacoustic cell, the frequency response of a circular plate of a two-layer sample (silicon + TiO_2_). Amplitudes and phases of the measured response (red stars) are shown in Figure 1. By removing the influence of the measuring chain (measuring instruments, especially detectors), corrected amplitudes and phases (black line) are obtained which can be analyzed by Equations (1)–(4) by the standard fitting method. The results of such analysis of the corrected signal give values of silicon (*l*_1_ = 30 μm), which corresponds to standard silicon substrate (thin plate) thicknesses, titanium-dioxide (*l*_2_ = 500 nm), which corresponds to standard thin-film thicknesses, and radius *R* = 3 mm, while other parameters correspond to the parameters from Table 1, with an error of 5%. The corrected signals from Figure 2 are further presented in our networks and the results of their prediction are given in Table 6 and Table 7. The relative error in these tables is the result of comparing network predictions and standard fitting of the existing theoretical model.

Based on the results of the prediction by neural networks NN1–3, (Table 6), the most accurate network is NN2 in the prediction of the thermal expansion coefficient $\alpha_{T1}^{\mathrm{NN}2}$ of a thin-film TiO_2_, with a relative (%) error <1%, while the precision in the prediction of the thermal diffusivity $D_{T1}^{\mathrm{NN}3}$ and thickness $l_{1}^{\mathrm{NN}1}$ is with relative (%) errors <5%.

In the simultaneous prediction of the parameters of thickness $l_{1}^{\mathrm{NN}4}$, thermal expansion coefficient $\alpha_{T1}^{\mathrm{NN}4}$ and thermal diffusivity $D_{T1}^{\mathrm{NN}4}$ (Table 7), the NN4 network gives satisfactory results comparable to the prediction results of NN1–3.

Despite the expectations based on the consideration of the theoretical model, which is reflected in the small visual difference of the amplitude characteristics and stratification of signal phases in the high-frequency part (1–20) kHz, neural networks based on the coupled amplitudes and phases in the frequency domain (20–20 k) Hz can determine the parameters of the thin-layer TiO_2_. The results of neural networks show that more precise and accurate results are obtained in networks in which multiple parameters are determined at the same time (Table 3 and Table 5) than in networks in the prediction of individual parameters (Table 2 and Table 4). This conclusion is also valid for the prediction of the thin-film parameter from the experimental results, where the reduction of the relative % error in the prediction of the network NN4 in relation to NN1–3 is observed, which can represent one of the methods of optimizing the work of networks in prediction the parameter of thin-films. It should be noted that the derived model is made for the expected ranges that each of the three parameters of the thin layer can have. If some of the parameters are outside this range, e.g., thickness of the thin-film, it could lead to incorrect determination of all three parameters of the thin-film using the proposed model.

This consideration is particularly valid due to the analysis of a thin layer of TiO_2_ placed on a well-characterized substrate, in this case, silicon. The method of characterization of TiO_2_ developed in this way can be applied and analyzed on other well-characterized optically transparent and non-transparent substrates. By applying TiO_2_ to optically transparent substrates, and by characterizing it, we obtain a suitable material for protecting the detectors of the measuring system.

## 7. Conclusions

The results presented in this paper indicate one very important fact—if in the measurement range, there is an influence of the thin-film on the total photoacoustic signal, neural networks easily can recognize these changes, even if they are negligibly small. Theoretical analyses of two-layer samples Si (substrate) and TiO_2_ (thin-film) showed relatively easy recognition of changes in the film of a thickness of ±5 nm, with the coefficient of thermal expansion of ±5 × 10^–8^ K^–1^ and coefficient of thermal diffusion of ±18.5 × 10^–8^ m^2^s^–1^.

In addition, it has been shown that neural networks for predicting thin-film parameters can be well-trained with a relatively small database, either to predict one or three parameters simultaneously. Furthermore, all networks give approximately the same accuracy of prediction in both theoretically generated signals and experimental data. Therefore, it can be recommended that, for the analysis of thin-films on different substrates, it is enough to form one network that simultaneously predicts several of its parameters instead of a separate network for determining each parameter.

## Figures and Tables

**Table 1:** Values of basic parameters used for PA simulation TiO_2_ thin-film deposed on Si substrate.

Parameters	Labels	Values
Air thermal diffusivity	*D_g_*[m^2^s^−1^]	2.0566 × 10^−5^
Air thermal conductivity	*k*_g_[W(mK)^−1^]	0.0454
Relaxation time of air	*τ*_g_[s]	2 × 10^−10^
Air adiabatic index	*γ*_g_	1.4223
Si Thermal diffusivity	*D_T_*_2_[m^2^s^−1^]	9 × 10^−5^
TiO_2_ Thermal diffusivity	*D_T_*_1_[m^2^s^−1^]	3.7 × 10^−6^
Si Thermal conductivity	*k*_2_[Wm^−1^K^−1^]	150.0
TiO_2_ Thermal conductivity	*k*_1_[Wm^−1^K^−1^]	11.0
Si Thermal expansion coefficient	*α_T_*_2_[K^−1^]	2.6 × 10^−6^
TiO_2_ Thermal expansion coefficient	*α_T_*_1_[K^−1^]	1.1 × 10^−5^
Si absorption coefficient	*β*_2_	2.58 × 10^5^
TiO_2_ absorption coefficient	*β*_1_	1.8 × 10^5^
Si reflexing coefficient	*R*_2_	0.3
TiO_2_ reflexing coefficient	*R*_1_	0.2
Si Young’s modulus	*Ey*_2_	1.37 × 10^11^
TiO_2_ Young’s modulus	*Ey*_1_	1.0 × 10^11^
Si Poison coefficient	*v*_2_	0.35
TiO_2_ Poison coefficient	*v*_1_	0.30

**Table 2:** Relative (%) error prediction of TiO_2_ thin-film parameters on 4 test photoacoustic signals that are in step by NN1, NN2 and NN3 networks.

Type of Network	NN1	NN2	NN3
Base	1	2	3
Parameter	$l_{1}^{\mathrm{NN}1}$	$\alpha_{T1}^{\mathrm{NN}2}$	$D_{T1}^{\mathrm{NN}3}$
TiO_2_ film no.1	0.4060	0.1041	0.3424
TiO_2_ film no.2	0.1681	0.1270	0.1526
TiO_2_ film no.3	0.1414	0.0690	0.2317
TiO_2_ film no.4	0.0658	0.1583	0.0764
Relative % error	0.1953	0.1146	0.2008

**Table 3:** Relative (%) error prediction of TiO_2_ thin-film parameters NN4 on 4 signals from three bases “in-step” of training network.

Type of Network	NN4								
Base	1			2			3		
Parameters	$l_{1}^{\mathrm{NN}4}$	$\alpha_{T1}^{\mathrm{NN}4}$	$D_{T1}^{\mathrm{NN}4}$	$l_{1}^{\mathrm{NN}4}$	$\alpha_{T1}^{\mathrm{NN}4}$	$D_{T1}^{\mathrm{NN}4}$	$l_{1}^{\mathrm{NN}4}$	$\alpha_{T1}^{\mathrm{NN}4}$	$D_{T1}^{\mathrm{NN}4}$
TiO_2_ film no.1	0.7878	0.8782	0.4542	0.3579	0.1592	0.9610	0.2958	0.0951	0.0152
TiO_2_ film no.2	0.0130	0.2941	0.3980	0.4126	0.4990	0.0564	0.0165	0.1059	0.2139
TiO_2_ film no.3	0.0187	0.2002	0.1512	0.7414	1.1077	1.3738	0.0932	0.0016	03694
TiO_2_ film no.4	0.1578	0.0588	0.2811	0.4206	0.8822	0.8822	0.1298	0.1278	0.0663
Relat % error	0.2443	0.3578	0.3211	0.4831	0.6434	0.8183	0.1325	0.0831	0.1661

**Table 4:** Relative (%) error prediction of TiO_2_ thin-film parameters NN1-3 on 4 signals from three bases “out of step” of training network.

Type of Network	NN1	NN2	NN3
Parameter	$l_{1}^{\mathrm{NN}1}$	$\alpha_{T1}^{\mathrm{NN}2}$	$D_{T1}^{\mathrm{NN}3}$
TiO_2_ film no.1	2.4890	0.0186	0.0777
TiO_2_ film no.2	2.4584	0.0293	0.0927
TiO_2_ film no.3	5.4138	0.0011	0.2593
TiO_2_ film no.4	4.8427	0.0031	0.0116
Relative % error	3.8099	0.0130	0.1103

**Table 5:** Relative (%) prediction error of TiO_2_ thin-film parameters by NN4 for 4 signals “out of step”.

Type of Network	NN4								
Base	1			2			3		
Parameter	$l_{1}^{\mathrm{NN}4}$	$\alpha_{T1}^{\mathrm{NN}4}$	$D_{T1}^{\mathrm{NN}4}$	$l_{1}^{\mathrm{NN}4}$	$\alpha_{T1}^{\mathrm{NN}4}$	$D_{T1}^{\mathrm{NN}4}$	$l_{1}^{\mathrm{NN}4}$	$\alpha_{T1}^{\mathrm{NN}4}$	$D_{T1}^{\mathrm{NN}4}$
TiO_2_ film no.1	0.1184	0.0422	1.33552	0.0173	0.0081	0.0164	0.0070	0.0049	0.0245
TiO_2_ film no.2	0.0422	0.0116	1.3516	0.0055	0.0183	0.0153	0.0182	0.0140	0.0104
TiO_2_ film no.3	0.0066	0.0599	1.3880	0.0080	0.0058	0.0270	0.0097	0.0215	0.0104
TiO_2_ film no.4	0.1213	0.0044	1.3685	0.0781	0.0225	0.0520	0.0245	0.0238	0.0059
Relative% error	0.0721	0.0368	1.3658	0.0272	0.0137	0.0277	0.0149	0.0161	0.0128

**Table 6:** Parameters $l_{1}^{\mathrm{NN}1}$, $\alpha_{T1}^{\mathrm{NN}2}$ and $D_{T1}^{\mathrm{NN}3}$ obtained by prediction of NN1-3, with relative (%) errors are calculated according to the parameters obtained from standard photoacoustics techniques.

Parameter	$l_{1}^{\mathrm{NN}1}$	$\alpha_{T1}^{\mathrm{NN}1}$	$D_{T1}^{\mathrm{NN}3}$
NN exp prediction	4.8018 × 10^2^ nm	1.0955 × 10^−5^ K^−1^	3.57913 × 10^−6^ m^2^s^−1^
relative (%) error	3.9644	0.4066	2.9372

**Table 7:** Parameters $l_{1}^{\mathrm{NN}4}$, $\alpha_{T1}^{\mathrm{NN}4}$ and $D_{T1}^{\mathrm{NN}4}$ obtained by prediction of NN4, with relative (%) errors are calculated according to the parameters obtained from standard photoacoustics techniques.

Parameter	$l_{1}^{\mathrm{NN}4}$	$\alpha_{T1}^{\mathrm{NN}4}$	$D_{T1}^{\mathrm{NN}4}$
NN4 exp prediction	4.8690 × 10^2^ nm	1.1166 × 10^−5^ K^−1^	3.7189 × 10^−6^ m^2^s^−1^
relative (%) error	2.6196	1.5106	0.5105

## Data Availability

The data that support the findings of this study are available from the corresponding author upon reasonable request.

## Appendix A. Temperature Distributions in Two-Layer Sample

Periodic temperature distributions in the thin-film (label 1 forTiO_2_) and substrate (label 2 for Si) illuminated by the modulated light source (Figure 1 and Figure 3) can be obtained by solving the thermal-diffusion equations in the form [21,31,59,62]:

(A1) $$\frac{\partial^{2}T_{1}\left(z,f\right)}{\partial z^{2}}-\frac{i\omega }{D_{T1}}T_{1}\left(z,f\right)=-\frac{1}{k_{1}}\beta_{1}\left(1-R_{1}\right)I_{0}e^{-\beta_{1}z}$$

and

(A2) $$\frac{\partial^{2}T_{2}\left(z,f\right)}{\partial z^{2}}-\sigma_{2}^{2}T_{2}\left(z,f\right)=-\frac{\varepsilon_{g}}{k_{2}\tau_{2}}n_{p2}\left(z,f\right)-\frac{\beta_{2}I}{k_{2}}\cdot \frac{\varepsilon -\varepsilon_{g}}{\varepsilon }e^{-\beta_{2}z}$$

where *ω =* 2*πf*, *f* is the modulation frequency, *I*_0_ is the incident light intensity, $I=\left(1-R_{1}\right)\left(1-R_{2}\right)e^{-\beta_{1}l_{1}}I_{0}$, *R*_1_ is the film reflection coefficient, *R*_2_ is the substrate reflection coefficient, $\sigma_{1}=\sqrt{i\omega /D_{T1}}$ is the film complex thermal diffusivity*, D_T_*_1_ is the film thermal diffusion coefficient, $\sigma_{2}=\sqrt{i\omega /D_{T2}}$ is the substrate complex thermal diffusivity*, D_T_*_2_ is the substrate thermal diffusion coefficient, *k*_1_ is the thin-film heat conduction coefficient, *k*_2_ is the substrate heat conduction coefficient, *β*_1_ is the film absorption coefficient, *β*_2_ is the substrate absorption coefficient, and *δn_p_*_2_(*z,f*) is the substrate photo-generated minority carrier dynamic density component (Equation (A2)).

The general solutions of Equations (A1) and (A2) can be written in the form [21,31,59,62]:

(A3) $$T_{1}\left(z,f\right)=A_{1}e^{\sigma_{1}z}+A_{2}e^{-\sigma_{1}z}+A_{3}e^{-\beta_{1}z},$$

and

(A4) $$T_{2}\left(z,f\right)=B_{1}e^{\sigma_{2}z}+B_{2}e^{-\sigma_{2}z}+B_{3}\delta n_{p}\left(z,f\right)+B_{4}e^{-\beta_{1}z},$$

where the constants *A*_3_, *B*_3_ and *B*_4_ are given as:

$$A_{3}=-\frac{\beta_{1}I_{0}(1-R_{1})}{k_{1}\left(\beta_{1}^{2}-\sigma_{1}^{2}\right)},\;B_{3}=-\frac{\varepsilon_{g}}{k_{2}\tau_{p2}\left(\sigma_{2}^{2}-\frac{1}{L_{2}^{2}}\right)},\;B_{4}=-\frac{\beta_{2}\left(1-R_{1}\right)\left(1-R_{2}\right)e^{-\beta_{1}l_{1}}I_{0}}{\varepsilon \left(\beta_{2}^{2}-\sigma_{2}^{2}\right)}\left(\frac{B_{3}}{D_{p2}}-\frac{\varepsilon -\varepsilon_{g}}{k_{2}}\right).$$

Here $L_{2}=\sqrt{\frac{D_{p2}\tau_{p2}}{1+i\omega \tau_{p2}}}$ is the complex minority carrier diffusion length, *D_p_*_2_ is the diffusion coefficient of minority carriers (holes *p* in the *n*-type substrate), and *τ_p_*_2_ is the bulk minority carrier lifetime. Constants *A*_1_, *A*_2_, *B*_1_ and *B*_2_ can be found solving the boundary conditions [21,31,59,62]:

$$(a)-k_{1}{\left.\frac{\partial T_{1}\left(z,f\right)}{\partial z}\right|}_{z=-l_{1}}=0,\;(b)\;T_{1}\left(0,f\right)=T_{2}\left(0,f\right),$$

$$(c)-k_{2}{\left.\frac{\partial T_{2}\left(z,f\right)}{\partial z}\right|}_{z=0}=s_{F}n_{p2}\left(0,f\right)\varepsilon_{g}-k_{1}{\left.\frac{\partial T_{1}\left(z,f\right)}{\partial z}\right|}_{z=0},$$

(A5) $$(d)-k_{2}{\left.\frac{\partial T_{2}\left(z,f\right)}{\partial z}\right|}_{z=l_{2}}=-s_{R}n_{p2}\left(l_{2},f\right)\varepsilon_{g},$$

where *s*_F_ and *s*_R_ are the substrate surface recombination speeds at the front (*z* = 0) and rear (*z* = *l*_2_) surfaces, respectively.

Based on our previous investigations, the analysis of the two-layer optical properties shows that the multiple optical reflections can be neglected in the Si substrate [31], but must be taken into account in the case of thin TiO_2_ film. This is the reason why the film reflection coefficient *R*_1_ is calculated here using [21,62]:

(A6) $$R_{1}=r_{F}+{\left(1-r_{F}\right)}^{2}r_{R}\cdot \frac{e^{-2\beta_{1}l_{1}}}{1-r_{F}r_{R}e^{-2\beta_{1}l_{1}}},$$

where *r*_F_ and *r*_R_ are the front and rear thin-film reflectivity coefficients, respectively.

## Appendix B. Two-Layer Sample Displacement along the Heat-Flow Axes

The *U_z,c_*(*r,z*) of the two-layer sample at the back surface, *z* = *l*_2_, important in transmission photoacoustic measurements, can be written in a general form as:

(A7) $$U_{z,c}\left(r,z\right)=\frac{C_{c}}{2}\left(R_{s}^{2}-r^{2}\right),\;c=TE,PE,$$

where *R_s_* is the sample radius and

(A8a) $$C_{\mathrm{TE}}=6\frac{A_{1}+A_{2}+E_{1}E_{2}\left[\alpha_{T1}l_{2}\left(2M_{T1}-l_{2}N_{T1}\right)+\alpha_{T2}l_{1}\left(2M_{T2}+l_{1}N_{T2}\right)\right]}{E_{1}^{2}l_{1}^{4}+E_{2}^{2}l_{2}^{4}+2E_{2}E_{1}l_{2}l_{1}\left(2l_{2}^{2}+3l_{2}l_{1}+2l_{1}^{2}\right)},$$

(A8b)
$$C_{\mathrm{PE}}=6d_{n}E_{2}\frac{\left[E_{1}l_{1}\left(2M_{n}+l_{1}N_{n}\right)+E_{2}l_{2}\left(2M_{n}-l_{2}N_{n}\right)\right]}{E_{2}^{2}l_{2}^{4}+E_{1}^{2}l_{1}^{4}+2E_{2}E_{1}l_{2}l_{1}\left(2l_{2}^{2}+3l_{2}l_{1}+2l_{1}^{2}\right)}.$$

Here $A_{1}=E_{1}^{2}l_{1}\left(2M_{T1}+l_{1}N_{T1}\right)\alpha_{T1},A_{2}=E_{2}^{2}l_{2}\left(2M_{T2}-l_{2}N_{T2}\right)\alpha_{T2},$ $E_{1}$ and $E_{2}$ are Young’s modulus of the film and substrate, respectively, $d_{n}$ is the coefficient of electronic deformation and $M_{T1}$, $M_{T2}$, $M_{n}$, $N_{T1}$, $N_{T2}$ and $N_{n}$ are defined as:

(A9) $$M_{T1}=\int_{-l_{1}}^{0}z\cdot T_{1}\left(z,f\right)dz,M_{T2}=\int_{0}^{l_{2}}z\cdot T_{2}\left(z,f\right)dz,M_{n}=\int_{0}^{l_{2}}z\cdot \delta n_{p2}\left(z,f\right)dz,$$

(A10) $$N_{T1}=\int_{-l_{1}}^{0}T_{1}\left(z,f\right)dz.N_{T2}=\int_{0}^{l_{2}}T_{2}\left(z,f\right)dz,N_{n}=\int_{0}^{l_{2}}\delta n_{p2}\left(z,f\right)dz,$$

where $T_{1}(z,f)$ is the temperature in the thin-film and $T_{2}(z,f)$ is the temperature in the substrate and $\delta n_{p2}\left(z,f\right)$ is the photo-generated minority carrier density. The $M_{T1}$, and $M_{T2}$ are the first moments of the temperature change, and the $M_{n}$ is the first moment of the photo-generated minority carriers change along the *z*-axis. The $N_{T1}$ and $N_{T2}$ are the average temperature changes and $N_{n}$ is the average photo-generated minority carriers change along the *z*-axes [21,31,59,62].
